# Acupuncture and moxibustion for cancer-related psychological disorders: A protocol for systematic review and meta-analysis

**DOI:** 10.1097/MD.0000000000028860

**Published:** 2022-03-11

**Authors:** Yan Jiang, Dan Liang, Yadi He, Jing Wang, Guixing Xu, Jun Wang

**Affiliations:** a Affiliated Hospital of Chengdu University of Traditional Chinese Medicine, Chengdu, People's Republic of China; b Acupuncture and moxibustion college, Chengdu University of Traditional Chinese Medicine, Chengdu, People's Republic of China; c Teaching Hospital of Chengdu University of Traditional Chinese Medicine, Chengdu University of Traditional Chinese Medicine, Chengdu, People's Republic of China.

**Keywords:** acupuncture and moxibustion, anxiety, cancer, depression, meta-analysis

## Abstract

**Introduction:**

Cancer-related psychological disorders (CRPD) with high incidence are often underdiagnosed and undertreated. Although, some studies suggested that acupuncture and moxibustion (AM) are effective and safe for CRPD, lacking strong evidence, for instance, the relevant systematic review, meta-analysis, and randomized control trial (RCT) of a large sample, multicenter, makes the effects and safety remain uncertain. The aim of protocol is to evaluating the RCTs of AM for CRPD to verify the association of AM with the improvement of CRPD.

**Methods and analysis:**

Eight electric databases (4 English databases and 4 Chinese databases) will be searched from inception to Mar. 2022. There will be no restrictions on the category of the language. The RCTs of AM for CRPD unlimited to any type of cancer will be included. Depression and anxiety scores will be the primary outcome indicators. Two researchers will independently complete study selection, evaluate the risk of bias, and extract the data. The RevMan 5.2 software will be used to conduct data synthesis using the random-effects model. The weighted mean differences or standardized mean differences with 95% CIs will be used to present the results of measurement data, and the risk ratios with 95% CIs will be used to express the counting data. Additionally, we will use the Grading of Recommendations Assessment, Development, and Evaluation to assess evidence quality.

**Main results:**

The results of the meta-analysis will be presented with tables and figures.

**Ethics and dissemination:**

The results of this meta-analysis and meta-regression will be disseminated via publication in peer-reviewed journals and will be published at relevant conferences. The data to be used will not contain individual patient data; therefore, there is no need to worry about patient privacy.

**PROSPERO registration number:**

CRD42020177219.

## 1 Introduction

Cancer-related psychological disorders (CRPD) can be defined and measured by patient self-reporting,^[1]^ although there is still no consensus of a clear and consistent definition of CRPD.^[2]^ The National Comprehensive Cancer Network defines CRPD as a multifactorial, unpleasant psychological (cognitive, behavioral, and emotional), social, and mental-emotional experience that may interfere with the ability to effectively respond to cancer, physical symptoms, and treatment. It is a continuum, from normal feelings (common fragility, sadness, and fear) to potentially disabling problems such as depression, anxiety, panic, social isolation, existence, and mental crisis.^[1]^ Approximately one-third to one-fourth of cancer patients have experienced CRPD during the disease, including anxiety or depression.^[3–7]^ These conditions are related to poor health-related quality of life (QOL)^[8,9]^ and poor survival rates.^[10]^ Depression can reduce the patients’ compliance with medical treatment regimens^[11]^ and make them more prone to fatigue after radiotherapy and chemotherapy.^[12,13]^ The diagnosis of CRPD is more difficult. Unlike the clinical manifestations of other psychological disorders, patients usually do not exhibit a depressed mood and are not aware of their anxiety and depression. Therefore, it is difficult to describe this feeling. In addition, in clinical practice, oncology nurses, and physicians fail to identify the suffering of cancer patients,^[14,15]^ and many patients’ problems may not be recognized; therefore, they remain largely untreated.^[16,17]^

Medication and complementary alternative therapies are the main treatments of CRPD.^[18–20]^ Acupuncture and moxibustion (AM) is part of the complementary alternative therapies, and well-known for treating pain, it is also designed to treat various physical and emotional disorders as an independent and complex medical system an extension of traditional Chinese medicine.^[21]^ The general public is increasingly using AM to treat various diseases; however, its use is particularly common among cancer patients and survivors.^[22–25]^ At present, evidence supports the use of AM to treat nausea and vomiting associated with cancer treatment as well as to treat hot flashes in cancer patients and survivors, leukopenia caused by chemotherapy, fatigue after chemotherapy, and xerostomia caused by radiation.^[25,26]^ Besides, increasing researches^[25,27–30]^ have suggested that AM might be useful for the treatment of commonly occurring cancer-related psychological symptoms. The pharmacological treatment of prevalent symptoms, such as anxiety, depression, and sleep disturbance can contribute to the high chemical burden already carried by cancer patients, creating additional side effects. As a result, patients and providers are interested in evidence-based nonpharmacologic alternatives, such as AM for these symptoms.

However, the effectiveness and safety of AM for CRPD are inconsistent in different studies.^[18,31–38]^ Therefore, we have suggested three key questions regarding AM treatment for CRPD:

1. Is AM effective for treating CRPD in a clinical setting?
2. Are there important factors affecting the efficacy of AM?
3. In the clinical practice of AM treatment for CRPD, has the specific effect of AM treatment changed compared with sham AM or other controls?

To address those key issues, we designed this systematic review and meta-analysis study protocol to evaluate the effectiveness and safety of AM for CRPD.

## 2 Methods

### 2.1 Criteria for considering studies for this review

#### 2.1.1 Types of studies

We will include RCTs randomly divided the subjects into 2 groups, regardless of whether used the blind method or not. Multiple arms trials fit in the above criteria are eligible. The data of the first period of crossover trials will also be included.

#### 2.1.2 Types of participants

We defined CRPD as cancer patients with depression or anxiety, ignoring the types of cancer and the reasons for depression and anxiety.

#### 2.1.3 Types of interventions

We defined AM as the needle piercing the acupoint, moxa-burned skin of the acupoint, acupressure of the acupoints, electropuncture, auricular needle, and transcutaneous electrical stimulation on acupoints. The control group will be defined as those receiving psychotropic medication, psychotherapy, or other non-AM therapy (e.g., sham AM).

### 2.2 Types of outcome measures

#### 2.2.1 Primary outcomes

Improvement of anxiety and depression: Hamilton Anxiety and Depression questionnaire score, Center for Epidemiological Studies-Depression, Generalized Anxiety Disorder 7, State–Trait Anxiety Inventory, Self-rating Depression Scale, and Self-rating Anxiety Scale.

#### 2.2.2 Secondary outcomes

1. Improvement of sleep disturbance: Pittsburgh Sleep Quality Index, sleep impairment index, the Leeds sleep scale, and Athens insomnia scale
2. Quality of life score
3. Side effects and serious side effects

### 2.3 Search methods for identifying studies

#### 2.3.1 Electronic searches

We will search the PubMed, Embase, Cochrane Library, Web of science, China National Knowledge Infrastructure, Wanfang Data Knowledge Service Platform, Chinese Biomedical Literature Database, and VIP database for Chinese Technical Periodicals from database inception to March 2022. The search strategy contains 3 components: study design (RCTs), disease (cancer-related depression or anxiety), and intervention (acupuncture, electropuncture, moxibustion, acupressure, etc.). The search strategy of PubMed is presented in Table 1. The results of the search will be imported to the endnote software.

**Table 1:** Illustrate the search strategy of PubMed.

Appendix I Search strategy used in Pubmed database	
Number	Search terms
#1	Randomized controlled trial [All Fields]
#2	Controlled clinical trial [All Fields]
#3	Randomized [All Fields]
#4	Randomised [All Fields]
#5	Placebo [All Fields]
#6	Randomly [All Fields]
#7	Trial [All Fields]
#8	Groups [All Fields]
#9	or/#1-#8
#10	Cancer OR carcinoma OR neoplasms [Title/Abstract]
#11	Acupuncture [Title/Abstract]
#12	Acupuncture therapy [Title/Abstract]
#13	Electroacupuncture [Title/Abstract]
#14	Electroacupuncture therapy [Title/Abstract]
#15	Manual acupuncture [Title/Abstract]
#16	Moxibustion [Title/Abstract]
#17	Acupoint [Title/Abstract]
#18	Auricular acupoint [Title/Abstract]
#19	Acupressure [Title/Abstract]
#20	Warm acupuncture [Title/Abstract]
#21	or/#11–#20
#22	#9 AND #10 AND #21

#### 2.3.2 Searching other resources

We will examine the existing systematic reviews or meta-analyses of AM for cancer to identify possible trials. In addition, the International Clinical Trials Registry Platform, Chinese clinical registry, and National Institutes of Health clinical registry Clinical Trials will be searched to find unpublished or ongoing trial data.

### 2.4 Data collection and analysis

#### 2.4.1 Selection of studies

**Figure 1:**
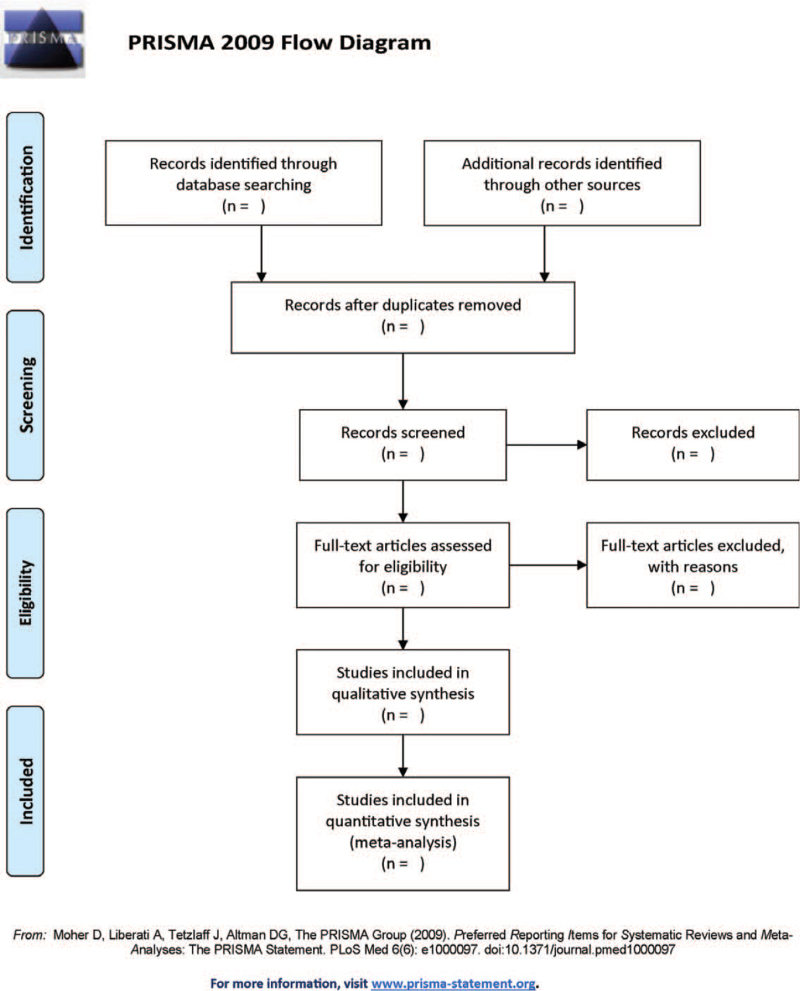
Illustrate the process and results of studies selection.

Two researchers will independently screen articles based on the inclusion and exclusion criteria. First, duplicate research will be eliminated according to the title and abstract. Then, based on the title and abstract of the study, studies not meeting the criteria will be excluded. Then, the full text of the included studies will be downloaded and read to determine whether the studies meet the inclusion criteria. Studies derived from other search results will also be screened according to the screening criteria. After research screening, the two researchers will cross-check the research results and decide on inconsistent results. If an agreement still cannot be reached, the decision will be made by a third party. The process and results are presented in Figure 1.

#### 2.4.2 Data extraction and management

Before data extraction, we will discuss the formulation of a specific data extraction program and use Excel 2016 to form a standard data extraction table. Data extraction will be independently completed by 2 researchers. Cross-checks will be conducted to check the original text against the differences to ensure data accuracy. The data required for the research include the following categories:

1. The general characteristics of the research, including the author, title, publication time, and journal of the research.
2. The general situation of CRPD patients in the study, including the sample size of the study, average age, gender, type of cancer, and the mental state of the patient before treatment.
3. The general situation of the research design, including the method of random grouping, blinding method, distribution of shadows, etc.
4. Relevant data of outcome indicators, including primary outcome and secondary outcome indicators.

#### 2.4.3 Assessment of risk of bias in included studies

The bias risk assessment included in the study will be independently completed by two researchers who will then cross-check the assessment results, discuss any disputes, and then make the final decision. If a consensus cannot be reached, we will invite a third expert to make an arbitrary decision. The bias risk assessment method is based on the Cochrane Collaboration's risk of bias tool.^[39]^ It mainly evaluates the use of hidden allocation, the use of blind methods, whether the data of the research results are complete, whether there is selective reporting, and other sources of risk of bias using random allocation methods. The evaluation results of each aspect are evaluated as correct (complete), incorrect (incomplete), and unclear.

#### 2.4.4 Measures of treatment effect

To address the clinical effect difference between the intervention and control groups, the anxiety and depression scale after treatment and the end of follow-up will be used as primary outcomes. Quality of sleep score, QOL, and side effects after treatment and at the end of follow-up will be used as secondary outcomes. For these continuous outcomes, the mean difference and SDs will be extracted and calculated as effect estimates.

#### 2.4.5 Unit of analysis issues

The results obtained from different evaluation scales cannot be directly used for data synthesis; therefore, we will divide the outcome indicators into depression rating scales, anxiety rating scales, sleep quality rating scales, and QOL rating scales. We will unify all evaluation data via the method of proportional conversion. The depression rating scale will be unified as the Hamilton depression scale scoring standard, the anxiety rating scale will be unified as the Hamilton anxiety scale, the sleep quality rating scale will be unified as the Pittsburgh Sleep Quality Index, and QOL will be unified as the Quality of Life Rating Scale.

#### 2.4.6 Dealing with missing data

If the study meets the inclusion criteria, but the data is incomplete, we will contact the author using the contact information provided in the study to obtain missing data. If data are not available, we will replace the data according to the method of multiple meta-analyses. If it is still not possible, we will only conduct a descriptive synthesis of the study.

#### 2.4.7 Assessment of heterogeneity

Before data analysis, the χ^2^ test will be used to assess the heterogeneity of the study. If *I*^2^ is less than 30%, the heterogeneity of the study will be defined as small. If *I*^2^ is greater than 30% and less than 60%, the degree of heterogeneity of the study will be defined as moderate. If *I*^2^ is greater than 60%, the degree of heterogeneity of the study will be defined as great, and we will not recommend or synthesize the data of the research results. When *I*^2^ is greater than 30%, we will use subgroup analysis, meta-regression, and other methods to find the source of heterogeneity.

#### 2.4.8 Assessment of reporting biases

If the number of included studies is greater than or equal to 10, we will draw a funnel chart to assess whether there is publication bias.

#### 2.4.9 Data synthesis

We will use the RevMan 5.2 software with the randomized effect model to conduct the data synthesis. This process will strictly follow the Cochrane Handbook for Systematic Reviews of Interventions. The risk ratios with 95% CIs and weighted mean differences or standardized mean differences with 95% CIs will be used to present the data synthesis outcome of dichotomous and continuous data, respectively. If there are fewer than three studies included in the final study, we will only conduct a narrative review and not merge the data.

#### 2.4.10 Subgroup analysis and investigation of heterogeneity

We will conduct subgroup analysis according to primary and secondary outcomes to detect possible heterogeneity. The following four subgroup analysis will be investigated:

1. The types of cancer.
2. Whether CRPD patients are undergoing radiotherapy and chemotherapy when receiving AM.
3. The types of AM, mainly divided into with and without needle piercing into the skin of the acupoint.
4. Western studies vs Chinese studies.

#### 2.4.11 Sensitivity analysis

To confirm the robustness of our research results, we will conduct sensitivity analysis based on the different levels of bias included in the study. To evaluate the internal validity of the study or the appropriateness of the treatment, we will then use the meta package and leave-out functions to remove biased high-risk studies, biased risk ambiguous studies, and biased low-risk studies.

#### 2.4.12 Summary of evidence

We will use the Grading of Recommendations Assessment, Development, and Evaluation (GRADE) method to assess the quality of evidence and provide a summary table of evaluation results. The summary table of the research results will be generated by the GRADE software [GRADEpro or GRADEpro GDT (www.gradepro.org)]. The content of the survey results summary table (the main results that are important to patients and decision-makers) will be determined by the above review team and where possible, relative and absolute measures of impact will be provided. To assess the quality of evidence, the grading method will classify the quality of evidence according to the results as high, medium, low, or very low. Evidence is categorized and downgraded based on concerns about bias, imprecision, inconsistency, discontinuity, or risk of publication bias. It can also be escalated through a large effect size, mixed negative effects, and dose-response relationships. The evaluator will downgrade or upgrade the evidence according to the scoring guidelines in Chapter 1134 of the Cochrane handbook (Cochrane handbook).

### 2.5 Ethics and dissemination

The results of this meta-analysis and meta-regression will be disseminated via publication in peer-reviewed journals and will be published at relevant conferences. The data to be used will not contain individual patient data. Therefore, there is no need to worry about patient privacy.

## 3 Discussion

The incidence of tumors has increased over the years, resulting in great pain to patients and families.^[40]^ Most cancer patients have bad emotions due to concerns regarding disease prognosis; medical costs; and side effects on the body caused by surgery, radiotherapy, and chemotherapy.^[12,41]^ Patients with tumors are affected by a variety of adverse emotions, resulting in decreased treatment compliance and immune function, which are not conducive to the control of the disease.^[18]^ Finding effective treatment options for CRPD is extremely important. AM is a part of the complementary alternative therapy that originated in China thousands of years ago. In acupuncture, a thin and strong metal needle is inserted into a specific anatomical part of the skin, and moxibustion is used as the burning-moxa to stimulate the acupuncture points or specific parts of the body. AM has been widely used to relieve nausea,^[42–44]^ vomiting,^[42–44]^ pain,^[45–48]^ insomnia,^[47,48]^ fatigue,^[49,50]^ and other symptoms^[51]^ in cancer patients. It has also been used to treat primary anxiety and depression, and its efficacy has been proven.^[34,52–56]^ The purpose of this study is to explore whether AM is also effective and safe for the treatment of CRPD. If this study proves that AM is effective and safe for cancer patients, it will benefit clinicians and patients and help them overcome cancer with a good mental state.

## Author contributions

**Conceptualization:** Dan Liang.

**Funding acquisition:** Jun Wang.

**Methodology:** Yadi He.

**Writing – original draft:** Yan Jiang, Dan Liang.

**Writing – review & editing:** Jing Wang, Jun Wang, Guixing Xu.
